# Endothelial Cells as Active Lipid Gatekeepers: Vascular Control of Lipid Handling and Metabolic Homeostasis

**DOI:** 10.3390/nu18071095

**Published:** 2026-03-29

**Authors:** Takeshi Kanda, Hidonori Urai

**Affiliations:** 1 Division of Nephrology, Department of Internal Medicine, Faculty of Medicine, Shimane University, Izumo 693-8501, Shimane, Japan; 2 The Center for Integrated Kidney Research and Advance (IKRA), Faculty of Medicine, Shimane University, Izumo 693-8501, Shimane, Japan; 3 Division of Nephrology, Endocrinology and Metabolism, Department of Internal Medicine, Keio University School of Medicine, 35 Shinanomachi, Shinjuku-ku, Tokyo 160-8582, Japan; h-urai@keio.jp

**Keywords:** endothelium, peroxisome proliferator-activated receptor γ, GHS-R, GPIHBP1, lipoprotein lipase, CD36, adipose tissue, skeletal muscle, heart, liver sinusoidal endothelium

## Abstract

Endothelial cells have emerged as critical peripheral nutrient sensors that actively regulate systemic lipid metabolism rather than serving as passive conduits. Endothelial peroxisome proliferator-activated receptor γ maintains redox balance, supports nitric oxide-dependent perfusion, and preserves insulin sensitivity during high-fat feeding, while ghrelin signaling through endothelial GHS-R promotes triglyceride clearance and lipid uptake into white adipose tissue through an endothelial peroxisome proliferator-activated receptor γ-dependent program. These pathways reveal that the endothelium integrates hormonal and metabolic cues to tune lipid trafficking, vectorial fatty acid delivery, and depot-specific energy storage. The concept that the endothelial phenotype, rather than circulating lipid levels alone, determines organ-level lipid exposure reframes endothelial lipid sensing as a key regulator of whole-body metabolic homeostasis. Understanding how endocrine and transcriptional pathways shape endothelial lipid handling may reveal new therapeutic targets for the treatment of obesity, dyslipidemia, and related metabolic diseases.

## 1. Introduction

**Figure 1:**
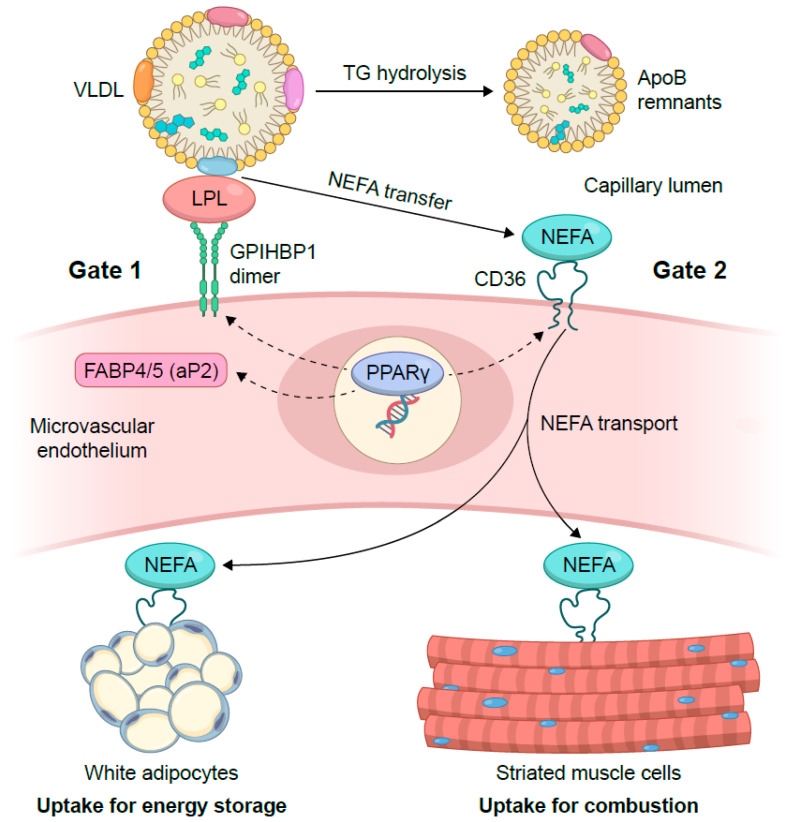
Two-stage endothelial lipid gate controlling tissue-specific lipid delivery.

A capillary-centric view of metabolism recognizes endothelial cells (ECs) as active gatekeepers that integrate hemodynamic, endocrine, and nutrient cues to distribute exogenous lipids to the parenchyma. Free fatty acids (FAs) are predominantly cleared by four metabolically active organs—adipose tissue, skeletal muscle, liver, and heart—each of which relies on endothelial interfaces to regulate substrate entry. These tissues differ in their oxidative capacity and storage functions, making endothelial gates a critical determinant of how circulating lipids are partitioned between them. Because adipose tissue serves as the primary reservoir for lipid storage, its microvascular architecture exerts a particularly strong influence on systemic lipid buffering. At the endothelial interface, lipid handling is coordinated by a set of specialized proteins that govern lipoprotein processing and fatty acid transfer. Lipoprotein lipase (LPL) is a triglyceride-hydrolyzing enzyme that catalyzes the breakdown of circulating triglyceride-rich lipoproteins, thereby generating free fatty acids for tissue uptake. Glycosylphosphatidylinositol-anchored high-density lipoprotein-binding protein 1 (GPIHBP1) is an endothelial transport protein that captures LPL in the interstitial space and displays it on the luminal capillary surface. Fatty acid translocase CD36 facilitates endothelial fatty acid uptake and initiates transcellular lipid transport, while fatty acid-binding proteins 4 and 5 (FABP4/5) support intracellular fatty acid trafficking within endothelial cells. In adipose tissue, single-cell and spatial atlases identify capillary EC clusters enriched for lipid-handling modules—including LPL, GPIHBP1, CD36, FABP4/5, and caveolar scaffolds—consistent with a specialized role in orchestrating postprandial lipid uptake [1]. In skeletal muscle and heart, substrate selection depends on the balance between FA and glucose delivery and oxidation, a balance constrained by the endothelial barrier [2]. In the liver, the sinusoidal endothelium (LSEC) is fenestrated and lacks a classic basement membrane, enabling macromolecular passage; defenestration and capillarization in metabolic dysfunction-associated steatotic liver disease (MASLD) impair this exchange and alter remnant handling [3]. As most extrahepatic tissues rely on continuous capillaries to regulate the entry of circulating lipids, ECs constitute a principal gate for FA- and triglyceride (TG)-rich lipoprotein (TRL)-derived flux into adipose tissue, skeletal muscle, and heart. In these organs, but not in the fenestrated liver sinusoid, the endothelium must execute a structured multistep lipid-handling program. An overview of this endothelial lipid-handling program is schematically illustrated in Figure 1. Endothelial lipid handling is organized through two sequential gates that regulate lipid processing across the endothelial barrier. At the luminal surface, lipoprotein lipase (LPL) is positioned and stabilized by glycosylphosphatidylinositol-anchored high-density lipoprotein-binding protein 1 (GPIHBP1), enabling efficient margination and hydrolysis of triglyceride-rich lipoproteins (Gate 1; Figure 1). Without this luminal display platform, LPL remains abluminally stranded, resulting in failed TRL processing and chylomicronemia. Following luminal lipolysis, fatty acids are transferred across endothelial cells via a transcellular transport gate (Gate 2; Figure 1), in which apical engagement of CD36 by long-chain fatty acids (LCFAs) initiates a caveolae-based endocytic program coupled to ceramide generation, culminating in basolateral export of FA-laden small extracellular vesicles to the parenchyma. These two gates are not independent; luminal lipolysis determines the supply of fatty acids to the apical membrane, while vesicular export governs how much of that supply ultimately reaches energy-consuming or storage tissues.

These two gates are dynamically regulated by coordinated transcriptional and endocrine inputs that couple endothelial lipid handling to physiological demand. Several circulating hormones and metabolites have been shown to influence endothelial lipid transport, including 3-hydroxyisobutyrate [4], angiopoietin-2 [5], and apelin [6], each acting in specific physiological contexts. Rather than providing a comprehensive survey of these signals, this review focuses on peroxisome proliferator-activated receptor γ (PPARγ) and ghrelin because they represent two complementary regulatory axes in endothelial lipid handling. PPARγ serves as a common downstream transcriptional hub that integrates metabolic cues within endothelial cells, whereas ghrelin directly conveys systemic energy demand to the endothelium through a defined endothelial receptor. This unique endocrine-to-endothelial linkage positions ghrelin as a key signal for coordinating lipid routing with organismal energy needs. In addition to lipid handling, endothelial cells also regulate insulin-stimulated glucose metabolism, underscoring their broader role in coordinating systemic nutrient distribution [7]. Collectively, endothelial PPARγ and ghrelin–GHS-R signaling represent complementary transcriptional and endocrine mechanisms that regulate Gate 1 and Gate 2, positioning the endothelium as an integrator of lipid and glucose distribution according to physiological demand.

Building on these concepts, the next part of the review focuses on the molecular systems that tune endothelial lipid handling. We first examine how PPARγ regulates the activity of both gates by coordinating perfusion, redox balance, and lipid-processing capacity. We then consider ghrelin–GHS-R signaling as an endocrine input that biases gate activity toward depot-specific lipid allocation through a PPARγ-dependent pathway. Through this framework, endothelial cells emerge as programmable regulators that integrate luminal lipolysis with transcellular fatty acid transfer to distribute fuel according to physiological demand.

Circulating triglyceride-rich lipoproteins are hydrolyzed at the luminal surface of capillaries by lipoprotein lipase (LPL) anchored by glycosylphosphatidylinositol-anchored high-density lipoprotein-binding protein 1 (GPIHBP1) (Gate 1). The resulting long-chain fatty acids (LCFAs) are subsequently transported across endothelial cells via a CD36-dependent, caveolae-mediated transcellular pathway and delivered to underlying parenchymal tissues (Gate 2). Transcriptional and endocrine signals, including endothelial PPARγ signaling, modulate perfusion, lipid-processing capacity, and depot-specific lipid allocation.

## 2. Endothelial PPARγ—Transcriptional Control of Vascular–Metabolic Coupling

PPARγ is a ligand-activated nuclear receptor that governs lipid handling, adipocyte differentiation, and systemic insulin sensitivity [8]. Traditionally recognized as the master regulator of adipogenesis and glucose–lipid homeostasis, PPARγ also shapes inflammatory tone, mitochondrial function, and redox balance across diverse cell types. Within the vasculature, its expression in ECs functions as a transcriptional integrator that links metabolic state to vascular function.

Over the past decade, EC PPARγ has emerged as a nutrient sensor. Mice lacking endothelial (and hematopoietic) PPARγ exhibit a paradoxical phenotype under high-fat feeding: reduced adiposity but elevated plasma TGs/free FAs, impaired vasodilation, and blunted responses to rosiglitazone [9]. Bone marrow reconstitution localizes these metabolic defects to the endothelium. Transcriptomic analyses identified an EC PPARγ program encompassing Cd36, Fabp4 (aP2), Gpihbp1, and redox/eNOS-coupling genes, indicating that PPARγ synchronizes capillary perfusion with lipid handling. Complementarily, EC-specific PPARγ modulates blood pressure under dietary stress and contributes to the antihypertensive actions of thiazolidinediones [10].

Mechanistically, endothelial PPARγ has been implicated in several interconnected functions based on genetic and pharmacological studies. First, it maintains an anti-inflammatory and antioxidant milieu that preserves eNOS coupling and NO bioavailability, enabling capillary recruitment and optimal hemodynamic access to TRLs and LCFAs [11]. Second, it induces lipid-handling components, including Cd36 and Fabp4/5, which expand FA recognition, binding, and intracellular routing [9]. Third, it upregulates Gpihbp1, thereby reinforcing luminal lipolysis by ensuring the proper localization and stability of LPL [12]. Fourth, it helps buffer acute dietary and hemodynamic stress, in part by limiting reactive oxygen species (ROS) that destabilize caveolar scaffolds and by constraining NF-κB-driven endothelial activation [13]. Together, these functions align perfusion (supply) with enzymatic platform capacity (processing) and vesicular throughput (delivery).

Beyond its canonical adipocyte-centric role, PPARγ also acts as a central molecular node in endothelial lipid handling. By orchestrating the transcription of genes governing FA uptake, intracellular trafficking, and luminal lipolytic competence, endothelial PPARγ enables capillaries to shape nutrient routing rather than passively receive circulating lipids. This dual identity—adipocytic and endothelial—places PPARγ at the core of whole-body lipid allocation. Within this framework, EC PPARγ functions as a metabolic integrator and gatekeeper that aligns tissue perfusion with lipid delivery and storage, positioning it as a master coordinator of systemic lipid flux across multiple compartments.

Additional considerations reinforce this centrality. Luminal lipolysis is jointly governed by GPIHBP1-mediated LPL stabilization and its antagonism by angiopoietin-like protein 4 (ANGPTL4), a secreted inhibitor of lipoprotein lipase that limits triglyceride hydrolysis in a tissue- and nutrient-dependent manner [14]; fluctuations in ANGPTL4 may dictate the extent to which PPARγ-driven Gpihbp1 induction improves TRL processing. Similarly, standardized profiling of EC-derived extracellular vesicles, particularly their CD36/ceramide cargo, provides a practical readout of transcellular throughput and helps distinguish luminal from vesicular bottlenecks. Single-cell atlases further show that adipose, muscle, and cardiac microvascular beds differ in the abundance of EC clusters enriched for lipid-handling modules [1,15], implying that the potency of PPARγ-dependent routing varies across tissues. Finally, by maintaining eNOS–NO coupling and repressing NF-κB-mediated inflammation, EC PPARγ preserves capillary recruitment, providing a mechanistic bridge between transcriptional tuning and hemodynamic access. Collectively, these insights consolidate endothelial PPARγ as a unifying regulator that matches metabolic supply with vascular delivery.

## 3. Ghrelin–Endothelial GHS-R—Endocrine Encoding of Storage Signals

Although the metabolic actions of ghrelin have long been attributed primarily to its central orexigenic pathways, accumulating evidence demonstrates that its peripheral endothelial actions constitute a distinct and physiologically meaningful branch of its signaling network. Ghrelin is a stomach-derived acylated peptide hormone that serves as the only known peripherally produced orexigenic signal that rises before meals and stimulates appetite. Its effects on feeding and weight gain are mediated predominantly through central mechanisms, particularly via activation of hypothalamic NPY/AgRP neurons and modulation of mesolimbic reward pathways that enhance food-seeking behavior. In addition to initiating hunger, ghrelin promotes positive energy balance by increasing meal size, reducing energy expenditure, and reinforcing the motivational salience of food. Through these centrally orchestrated actions, ghrelin functions as a key integrator of nutritional status, energy homeostasis, and body weight regulation [16].

Our recent study revealed a complementary endothelial branch of ghrelin biology. We have previously shown that ghrelin elevates adiposity and accelerates TG clearance in wild-type mice, but not in GHS-R-null animals [17]. Selective restoration of GHS-R in the ECs of knockout mice rescues WAT lipid uptake and LPL activity, thereby assigning sufficiency to the endothelial receptor. In cultured ECs, ghrelin increases FA uptake and upregulates lipid transport transcripts, whereas these effects are lost with GHS-R deficiency and attenuated by PPARγ knockdown, placing PPARγ downstream of endothelial GHS-R. This circuit transduces endocrine information regarding energy needs into a vascular instruction set that promotes safe storage when appropriate.

Historically, ECs have been viewed primarily as sensors of local mechanical cues, such as shear stress, hydrostatic forces, and microenvironmental gradients, which modulate their barrier, vasomotor, and metabolic functions. However, accumulating evidence demonstrates that ECs are also hormonally responsive, integrating endocrine signals with local hemodynamic inputs to recalibrate lipid handling and coordinate systemic energy distribution. The endothelial expression of GHS-R across multiple vascular beds further supports the feasibility of a direct vascular pathway through which ghrelin influences nutrient routing.

Moreover, this endocrine responsiveness is not only theoretical but also functionally sequential. Our work shows that ghrelin acts not only through its well-established central pathways but also directly on ECs, where GHS-R activation enhances lipid uptake and increases adipose tissue mass. This peripheral endothelial mechanism expands the canonical view of ghrelin biology and demonstrates that vascular cells participate in the orchestration of nutrient allocation and depot-specific fat accretion.

To become biologically active, ghrelin requires octanoylation of its third serine residue, a unique posttranslational modification mediated by ghrelin O-acyltransferase [18]. This modification depends on the availability of medium-chain FAs, particularly octanoic acid, which serves as an essential acyl donor [19]. Because medium-chain FAs are absorbed rapidly and efficiently enter the ghrelin-acylating pathway, their availability effectively amplifies the orexigenic drive of ghrelin, enhancing appetite, body weight gain, and lipid accumulation [20]. These biochemical features provide a mechanistic bridge between nutrient composition and vascular lipid routing, implying that dietary medium-chain FAs modulate both the endocrine and endothelial branches of the ghrelin axis.

Collectively, these findings position ghrelin–GHS-R signaling as a vascular–endocrine interface that complements endothelial PPARγ. Whilst PPARγ tunes perfusion, redox balance, and luminal and vesicular lipid-handling capacity, ghrelin enhances storage-side readiness and depot-specific allocation. Together, these two pathways constitute a dual-input system through which the endothelium aligns lipid routing with organismal energy demands.

## 4. Two-Stage Lipid Gate: Luminal LPL–GPIHBP1 and Transcellular CD36–Caveolae

As schematically illustrated in Figure 1, endothelial lipid handling operates as a continuous two-stage process, beginning with luminal LPL–GPIHBP1-mediated lipolysis and culminating in CD36-dependent transcellular delivery of fatty acids to the parenchyma. LPL, synthesized by myocytes and adipocytes, functions exclusively in the capillary lumen, a topological challenge resolved by GPIHBP1, whose three-fingered LU domain binds the *C*-terminal lipid-binding region of LPL, whereas its intrinsically disordered acidic domain accelerates association and prevents unfolding of the LPL hydrolase domain [21,22]. Through these biochemical properties, GPIHBP1 captures LPL from the interstitium, ferries it across ECs, and displays it on the luminal surface, enabling efficient margination and hydrolysis of TRLs. In the absence of GPIHBP1, LPL becomes abluminal, intravascular TRL processing collapses, and severe hypertriglyceridemia ensues; structural studies further show that the acidic domain electrostatically sheaths a basic patch on LPL, preventing pathological interactions with heparan sulfate proteoglycans that would otherwise trap LPL on the incorrect side of the endothelium.

Once luminal lipolysis generates LCFAs, endothelial CD36 initiates a vectorial transport program that transfers these lipids to parenchymal tissues. LCFA binding to apical CD36 triggers Src-dependent phosphorylation of caveolin-1 at Y14 and local ceramide generation within caveolae, driving the fission of 80 to 100 nm vesicles that carry FA, CD36, and ceramide to the basolateral surface [23]. These vesicles are released as small extracellular vesicles, which deliver lipid cargo directly to underlying myotubes in Transwell systems. In vivo, suppression of exosome biogenesis reduces muscle FA uptake, increases circulating FA that persists within the vasculature, and lowers the glucose-recapitulating metabolic features of endothelial CD36 deficiency. Cell-specific knockout experiments further confirm that deletion of CD36 in ECs, but not in parenchymal cells, elevates fasting FA and postprandial TG levels while reducing FA uptake in the heart, skeletal muscle, and brown adipose tissue, establishing the endothelium as the primary gatekeeper for systemic FA entry [24].

Together, these mechanisms define a serial architecture for endothelial lipid handling: the luminal platform determines supply—how much FA becomes available at the apical membrane—while the vesicular export apparatus determines delivery—how much ultimately reaches energy-consuming or storage tissues. PPARγ strengthens both layers by improving perfusion, inducing Gpihbp1 expression, and maintaining caveolar integrity; ghrelin–GHS-R signaling increases depot-side demand and enhances endothelial processing capacity through PPARγ-dependent transcription. Microenvironmental factors, such as shear stress, glycocalyx thickness, caveola density, and capillary rarefaction, modulate throughput, explaining why identical circulating lipid levels can result in divergent organ exposure. Failure at any node produces a recognizable signature: luminal failure manifests as low soluble GPIHBP1 or post-heparin LPL mass or activity with elevated TG area under the curve; transcytosis overactivity appears as high EV ceramide and increased muscle PET FA uptake; and perfusion defects present with impaired flow-mediated dilation or peripheral arterial tonometry and reduced adipose tissue blood flow. This integrated framework clarifies how ECs orchestrate the spatial allocation of lipid substrates across tissues.

## 5. Vascular Bed-Specific Rules—Adipose, Skeletal Muscle, Heart, and Liver Sinusoids

Endothelial control of lipid routing is not uniform across the body (Table 1). Instead, each vascular bed deploys a distinct combination of luminal, transcellular, and perfusion mechanisms that reflect the metabolic mandates of the underlying tissue. Understanding these bed-specific endothelial programs is essential for predicting how identical circulating lipid levels can produce widely divergent organ exposures and for identifying which nodes are targetable in disease.

Across metabolic tissues, the architecture and specialization of the microvasculature dictate how effectively ECs govern lipid routing, and distinct vulnerabilities emerge as physiological demands change. In WAT, continuous but nonfenestrated capillaries sustain high lipid flux through robust luminal and transcellular machinery [28]. Obesity disrupts this system by inducing capillary rarefaction, endothelial inflammatory activation, and decreased adipose tissue blood flow, thereby weakening lipid buffering and promoting hypoxia and fibrosis. Therefore, restoring endothelial function requires coordinated reinforcement of perfusion through NO-dependent vasodilation, stabilization of the GPIHBP1–LPL luminal platform, and careful calibration of transcytosis to prevent ectopic spillover. These considerations are depot-specific [29]. Subcutaneous WAT typically preserves perfusion and buffering capacity better than visceral depots, establishing a therapeutic opportunity to steer lipid flux toward safer adipose beds when redistribution is required [30].

In skeletal muscle, oxidative fibers depend heavily on FA oxidation to sustain endurance [25]; however, they are susceptible to lipid-induced insulin resistance when oversupplied. Endothelial CD36 deletion lowers FA uptake into muscle and heart while raising plasma FA levels, illustrating that the endothelial step represents the principal choke point for FA entry [24]. Modulating the CD36–caveolae axis can therefore reduce lipotoxic load and improve glycemia, although such interventions must respect performance demands [23]. Synchronizing treatment with rest periods and monitoring substrate handling through PET-based FA uptake imaging can preserve exercise capacity.

Cardiac tissue further illustrates the delicate balance between supply and demand. The myocardium relies extensively on FA oxidation, and cardiomyocyte-derived LPL depends on GPIHBP1 for luminal positioning [25,26]. Insufficient LCFA delivery, which risks energetic shortfalls, or excessive delivery, which causes lipotoxic stress, impairs cardiac performance [25]. Precision approaches aim to stabilize the GPIHBP1–LPL interface while dynamically tracking myocardial substrate switching between FA and glucose via PET imaging and monitoring natriuretic peptides to optimize energetics in diabetes, HFpEF, and other high-demand states.

By contrast, the liver operates under fundamentally different endothelial constraints. The fenestrated LSEC allows efficient passage of remnants and macromolecules, enabling a distinct mode of lipid surveillance compared with continuous capillary beds. In MASLD, defenestration and capillarization reduce sinusoidal porosity and disrupt drug and lipid handling. Because LSECs do not rely on the GPIHBP1–LPL axis in the same manner as other tissues, hepatically focused strategies emphasize preserving fenestrae and maintaining scavenger receptor function, whereas systemic therapies reduce TG burden and prevent remnant overload.

Collectively, these bed-specific features highlight a central principle: lipid exposure is determined less by plasma concentrations than by endothelial phenotype, including structural integrity, recruitment capacity, luminal display, and vesicular throughput. These concepts are supported by a growing body of experimental and translational studies, although their clinical applicability remains to be established. Recognizing this heterogeneity reframes dyslipidemic disease not as a disorder of circulating lipids alone, but as a mismatch between routing capacity and tissue metabolic demand.

This perspective naturally sets the stage for future research aimed at determining how distinct modes of endothelial failure—luminal, transcellular, or perfusion-related—might be selectively targeted. By mapping these vulnerabilities to potentially actionable levers, subsequent studies may develop frameworks for correcting lipid misallocation at vascular points of control.

## 6. Conclusions

The endothelium, which is positioned at every interface where circulating nutrients meet the parenchyma, is a programmable gate for lipid routing. A two-stage architecture—luminal LPL–GPIHBP1 and transcellular CD36–caveolae—operates under transcriptional (PPARγ) and hormonal (ghrelin–GHS-R) control to match supply with demand. Failure at any node is measurable with composite biomarkers and imaging and can be repaired with mechanism-matched levers. This paradigm reframes dyslipidemia, insulin resistance, MASLD, and heart failure energetics as disorders of vascular nutrient logistics. Although therapeutic targeting of endothelial lipid routing is an emerging concept, early mechanistic studies suggest that perfusion control, luminal lipase stabilization, and vesicular FA export represent actionable levers requiring future validation. By endotyping patients, deploying short-cycle, mechanism-linked trials, and titrating EC-targeted therapies with objective readouts, lipid routing can become a controllable clinical parameter, similar to blood pressure, enabling a transition beyond glucose-centric care toward integrated vascular metabolic medicine.

## Figures and Tables

**Table 1:** Bed-specific endothelial differences in lipid handling across metabolic tissues.

Organ/Tissue	Endothelial Type	Key Molecular Signatures	Features of Lipid Handling	Pathophysiological Feature
White adipose tissue (WAT)	Continuous capillary	High GPIHBP1, high LPL, CD36 ↑, abundant caveolae, FABP4/5	Strong postprandial lipid buffering; efficient TRL processing and FA uptake	Obesity causes capillary rarefaction, reduced adipose tissue blood flow, impaired buffering, increased spillover
Skeletal muscle	Continuous capillary	CD36 ↑, caveolin-1 ↑, moderate FABP4/5	Relies heavily on FA transcytosis; FA delivery adjusts to contraction demands	Excess FA delivery → intramyocellular lipid accumulation → insulin resistance
Heart (Cardiac muscle)	Dense continuous capillary network	High LPL, GPIHBP1 ↑, CD36 ↑, high FA oxidation capacity	Constant FA-dominant energy metabolism; substrate delivery strongly depends on microvascular perfusion	Oversupply → lipotoxicity; undersupply → energetic deficit (e.g., HFpEF)
Liver (LSEC)	Fenestrated sinusoidal endothelium	Fenestrae, Stabilin-1/2, low CD36	Passive uptake of TRL remnants through fenestrae; minimal need for luminal lipolysis	MASLD leads to capillarization, loss of fenestrae, impaired remnant clearance

This table summarizes the structural and molecular features of endothelial cells in white adipose tissue, skeletal muscle, heart, and liver, highlighting how endothelial phenotype (continuous versus fenestrated architecture), expression of lipid-handling genes, and reliance on luminal versus transcellular fatty acid (FA) transfer determine organ-level lipid exposure [25,26,27]. These bed-specific traits underlie distinct metabolic functions, such as adipose lipid buffering, muscle FA uptake during contraction, cardiac fatty-acid-dominant energy metabolism, and hepatic remnant clearance, and help explain how endothelial dysfunction contributes to metabolic disease across tissues. TRL, triglyceride-rich lipoproteins; MASLD, metabolic dysfunction-associated steatotic liver disease; LSEC, liver sinusoidal endothelium.

## Data Availability

No new data were created or analyzed in this study.

## Abbreviations

The following abbreviations are used in this manuscript:

EC	Endothelial cell
FA	Fatty acid
LPL	Lipoprotein lipase
LSEC	Liver sinusoidal endothelium
MASLD	Metabolic dysfunction-associated steatotic liver disease
TG	Triglyceride
TRL	Triglyceride-rich lipoprotein
LCFA	Long-chain fatty acid
PPARγ	Peroxisome proliferator-activated receptor γ
NO	Nitric oxide
WAT	White adipose tissue
ANGPTL4	angiopoietin-like protein 4
ROS	reactive oxygen species
